# Weather sensitivity associated with quality of life in patients with fibromyalgia

**DOI:** 10.1186/s41927-021-00185-4

**Published:** 2021-05-10

**Authors:** Kazuhiro Hayashi, Kenji Miki, Noriyuki Hayashi, Ryota Hashimoto, Masao Yukioka

**Affiliations:** 1 Multidisciplinary Pain Center, Aichi Medical University, Nagakute, Japan; 2 Center for pain management, Hayaishi Hospital, Osaka, Japan; 3 Faculty of Health Science, Osaka Yukioka College of Health Science, Osaka, Japan; 4 Department of Psychiatry, Yukioka Hospital, Osaka, Japan; 5 Department of Pathology of Mental Diseases, National Institute of Mental Health, National Center of Neurology and Psychiatry, Kodaira, Japan; 6 Department Rheumatology, Yukioka Hospital, Osaka, Japan

**Keywords:** Fibromyalgia, Weather, Quality of life, Pain, Observation

## Abstract

**Background:**

Fibromyalgia is characterized by chronic widespread pain, and more than half of patients with fibromyalgia report that weather-related variables aggravate their symptoms. However, the differences in actual symptoms have not been measured between those with and without weather sensitivity. The present study aimed to investigate whether weather sensitivity associated with the minimal clinically important difference values of quality of life in patients with fibromyalgia, between those with and without weather sensitivity.

**Methods:**

Sixty-four consecutive outpatients with fibromyalgia on their first visit to our tertiary center were included. Weather sensitivity was measured using self-perceived symptoms. Pain intensity was measured using the 0–10 Numerical Rating Scale (NRS). Quality of life was measured using the Euro Quality of life-5 Dimensions-3 level (EQ-5D-3L) scale. The variables were subjected to univariable and multivariable analysis using the EQ-5D-3L scale.

**Results:**

The mean age of the patients was 50 years. Forty-eight patients (75%) were women. The mean EQ-5D-3L score was 0.55. Thirty-seven patients (58%) reported weather sensitivity. In univariable analysis, the welfare recipient, weather sensitivity, and NRS values were associated with EQ-5D-3L scale scores. In multivariable analysis, NRS value and weather sensitivity were independently associated with EQ-5D-3L scale scores. The NRS and EQ-5D-3L scale scores were significantly worse in those with weather sensitivity than those without weather sensitivity. The difference in NRS values was less than 1.5 points between groups. The differences in EQ-5D-3L scale scores were 0.16 points between groups.

**Conclusions:**

Weather sensitivity was significantly associated with quality of life in patients with fibromyalgia. There was an association with weather sensitivity and the minimal clinically important difference values of quality of life in patients with fibromyalgia. The presence of weather sensitivity could have a key role in the quality of life in patients with fibromyalgia.

**Supplementary Information:**

The online version contains supplementary material available at 10.1186/s41927-021-00185-4.

## Background

Fibromyalgia is a disease characterized by chronic widespread pain without a known cause or cure [1–4]. The prevalence of fibromyalgia is estimated to be approximately 2% in the general population [5–9]. The coexisting symptoms are sleep disturbance, fatigue, depression, and anxiety; however, these are not universal [1–4]. Clinical care of patients with fibromyalgia is uncertain in many areas. The routine care in patients should include a comprehensive assessment of quality of life, and not only specific symptoms [10, 11]. The Euro quality of life-5 Dimensions (EQ-5D) scale is a generic patient-reported quality of life measurement. The scale can be used for cost-effectiveness studies across diseases. It is a 5-item questionnaire that after appropriate weighting results in a scale ranging from − 0.11 to 1.00. Fibromyalgia has a negative impact on quality of life, with an EQ-5D scale score of 0.45–0.61 [12, 13].

Potential factors that aggravate fibromyalgia symptoms are weather-related variables, physical activity, emotional distress, and sleep disturbance [14, 15]. Specifically, weather-related variables are one of the major aggravating factors [14–19]. Weather-related variables are measured using self-perceived symptoms or local weather situations [14–25]. In recent patient population studies, weather-related variables were significantly associated with pain in fibromyalgia; however, the associations were too minor to be of any clinical significance [20–25]. Thus, a recent study hypothesizes that the relation of weather-related variables may be clinically significant at the individual level [25]. More than half of the patients reported that weather sensitivity aggravates pain; however, the differences in actual symptoms have not been measured between those with and without weather sensitivity [14–19].

The present study aimed to investigate whether weather sensitivity associated with the minimal clinically important difference values of quality of life in patients with fibromyalgia, between those with and without weather sensitivity.

## Methods

### Subjects

All methods were carried out in accordance with relevant guidelines and regulations. This study was approved by the Ethics Committee of Hayaishi Hospital, and all study participants provided written informed consent. The sample size was calculated using the G*Power software (version 3.1.9.2; Franz Faul, Kiel University, Kiel, Germany). Based on an effect size of 0.3, [20–25] the minimum number of subjects was estimated to be 64 for an α-level of 0.05, and a power (1 – β) of 0.80.

Data were retrospectively collected from medical records of consecutive outpatients with fibromyalgia during their first visit to our tertiary center. Inclusion criteria were: 1) age > 20 years; and 2) a diagnosis of fibromyalgia by a medical doctor, based on the American College of Rheumatology 2010 criteria [1, 26]. The diagnostic criteria consist of the Widespread Pain Index and the Symptom Severity Scale. Exclusion criteria were cancer-related pain, neurological disease, evidence of bone fractures, recent surgery within the past 6 months, or poor Japanese language comprehension. A total of 64 patients with fibromyalgia were included (from February 2017 to June 2018) in the analysis.

### Measures

Demographic data, including age, body mass index, sex, marital status, and welfare recipient were collected during the first visit to our tertiary center. Weather sensitivity, pain intensity, and quality of life were measured as described below.

Weather sensitivity was routinely taken for all patients in our institution. Weather sensitivity was measured using self-perceived symptoms [14–19]. Subjects were assigned into two categories, those “with weather sensitivity” and “without weather sensitivity”, based on their “Yes” or “No” responses to single question: “Does change in weather affect your pain?” [14, 16].

Pain intensity was measured using the 0–10 Numerical Rating Scale (NRS) [27]. The scale, ranging from 0 to 10, was obtained as an indicator of the average level of pain during the day. The scale labeled at the anchor points with 0 as “no pain” and 10 as “worst possible pain”. The minimal clinically important difference was 2 points in chronic pain [28, 29].

Quality of life was measured using the Euro Quality of life-5 Dimensions-3 level (EQ-5D-3L) scale, a generic scale used worldwide that assesses health in five dimensions: mobility, self-care, usual activities, pain/discomfort, and anxiety/depression [10, 11]. Each domain was assessed by a single question with three possible responses: no problems, some problems, or serious health problems. The combination of all possible dimensions and levels results in 243 unique health states. It can be converted into EQ-5D-3L scale scores ranging from − 0.111 to 1.000. Negative scores represent health states considered worse than being dead, 0 represents being dead, and 1.00 represents a state of full health. The minimal clinically important difference was 0.08 points in chronic pain [28, 29].

### Statistical analysis

All continuous data were expressed as means and standard deviations. The normality of the distribution was evaluated using the Shapiro-Wilk test for continuous variables. The correlation of EQ-5D-3L scale score with each variable was analyzed using the Pearson correlation coefficient test. The categorical variables included a dummy variable for the analysis. Multivariable analysis was used to investigate variables with *p* < 0.2 in the univariable analysis. Four variables were analyzed in the multivariable analysis for EQ-5D-3L scale score: marital status, welfare recipient, weather sensitivity, and the NRS values. Multicollinearity of the variables was also assessed (correlation coefficient < 0.9).

All data were statistically analyzed using the SPSS 26.0 J program, and *P* values < 0.05 were considered significant.

## Results

Patient characteristics are shown in Table 1. The mean age of the patients was 50 years. Of the 64 patients, 48 (75%) were women. The number of patients with weather sensitivity was 37 (58%). The mean NRS value was 5.8. The mean EQ-5D-3L score was 0.55.

**Table 1:** Patient characteristics (*n* = 64)

Age, y	50 (16)
Body mass index, kg/m^2^	22 (4)
Women, n (%)	48 (75%)
Married, n (%)	31 (48%)
Welfare recipient, n (%)	9 (14%)
Weather sensitivity, n (%)	37 (58%)
NRS	5.8 (1.7)
EQ-5D-3L scale	0.55 (0.18)

*EQ-5D-3L* Euro Quality of life-5 Dimensions-3 level, *NRS* Numerical Rating Scale

Data from continuous variables are shown as mean (standard deviation). Data from categorical variables are shown as number (%)

Correlations between the EQ-5D-3L scale scores and independent variables are shown in Table 2. The welfare recipient, weather sensitivity, and NRS value were significantly associated with EQ-5D-3L scale scores in univariable analysis.

**Table 2:** Correlation between EQ-5D-3L scale and the independent variables

	Correlation coefficient	*P* value
Age	0.083	0.512
Women	−0.123	0.333
Body mass index	−0.084	0.507
Married	0.224	0.076
Welfare recipient	−0.313	0.012*
Weather sensitivity	−0.438	< 0.001*
NRS	−0.522	< 0.001*

*EQ-5D-3L* Euro Quality of life-5 Dimensions-3 level, *NRS* Numerical Rating Scale

These data were analyzed using the Pearson correlation coefficient test. The categorical variables included a dummy variable for analysis. The welfare recipient, weather sensitivity, and NRS value were significantly associated with EQ-5D-3L scale scores in the univariable analysis. *Significance level was set at < 5%

The results of multivariable analysis for the EQ-5D-3L scale scores are shown in Table 3. The EQ-5D-3L scale score was significantly associated with the NRS value and weather sensitivity in multivariable analysis. Multicollinearity was not observed for any of the independent variables tested in the analyses.

**Table 3:** Multivariable analysis for EQ-5D-3L scale

Independent Variables	B	SE	Beta	*P* value	R^2^
NRS	−0.035	0.012	−0.344	0.004*	0.349
Weather sensitivity	−0.106	0.040	−0.301	0.010*	
Welfare recipient				0.065	
Married				0.651	

B, nonstandard regression coefficient; Beta, standardized regression coefficient; *EQ-5D-3L* Euro Quality of life-5 Dimensions-3 level, *NRS* Numerical Rating Scale, *R*^*2*^ Multiple correlation coefficient adjusted for degrees of freedom, *SE* Standard error

These data were analyzed using multivariable analysis. Multicollinearity of the variables was also assessed (correlation coefficient < 0.9). EQ-5D-3L scale score was significantly associated with the NRS value and weather sensitivity in multivariable analysis. *Significance level was set at < 5%

**Fig. 1:**
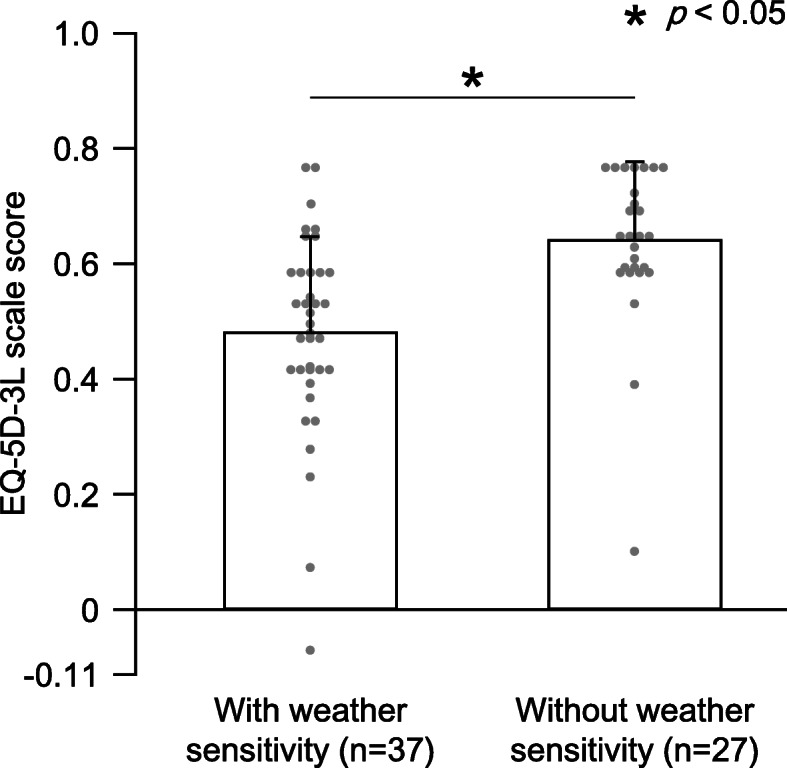
Difference of EQ-5D-3L scale between those with and without weather sensitivity. Values are means of the EQ-5D-3L scale score, and the error bar shows standard deviations. Dot plots show individual patient data. The EQ-5D-3L scale scores in those with weather sensitivity were significantly worse than those without weather sensitivity. * Significance level was set at < 5%.EQ-5D-3L, Euro Quality of life-5 Dimensions-3 level

Table 4 and Fig. 1 show a comparison between those with and without weather sensitivity. The NRS values and EQ-5D-3L scale scores were significantly worse in those with weather sensitivity than those without weather sensitivity. The difference in NRS values was less than 1.5 points between groups. The differences in EQ-5D-3L scale scores were 0.16 points between groups.

**Table 4:** Comparison between those with and without weather sensitivity

	With weather sensitivity (*n* = 37)	Without weather sensitivity (*n* = 27)	*P* value
Age, y	50 (15)	50 (17)	0.996
Body mass index, kg/m^2^	23 (4)	22 (4)	0.201
Women, n (%)	29 (78%)	19 (70%)	0.563
Married, n (%)	16 (43%)	15 (56%)	0.448
Welfare recipient, n (%)	5 (14%)	4 (15%)	> 0.999
NRS	6.3 (1.7)	5.0 (1.5)	0.001*
EQ-5D-3L scale	0.48 (0.17)	0.64 (0.14)	< 0.001*

*EQ-5D-3L* Euro Quality of life-5 Dimensions-3 level, *NRS* Numerical Rating Scale

These data were analyzed using t test or chi-square test. Data from continuous variables are shown as mean (standard deviation). Data from categorical variables are shown as number (%). The NRS value and EQ-5D-3L scale score were significantly worse in those with weather sensitivity than those without weather sensitivity. * Significance level was set at < 5%

## Discussion

The present study demonstrated that weather sensitivity was significantly associated with quality of life in patients with fibromyalgia. The findings suggested that the presence of weather sensitivity could have a key role in the quality of life in these patients.

Fibromyalgia induces pain and reduces the quality of life [12, 13]. Previous patient population studies suggested that associations with weather-related variables and pain were too small to be clinically significant in patients with fibromyalgia [20–25]. The minimal clinically important difference for NRS value is 2 points in chronic pain [28, 29]. The present study demonstrated that the difference in NRS values was less than 1.5 points between groups; this suggests that the association with weather sensitivity and pain was too small to be clinically significant, consistent with previous studies [20–25]. Meanwhile, the minimal clinically important difference for EQ-5D scale score is 0.08 points in chronic pain [28, 29]. The present study demonstrated that the differences in EQ-5D scale scores were 0.16 points between groups. The EQ-5D scale consists of mobility, self-care, activity, pain/discomfort, and anxiety/depression [10, 11]. Patients with fibromyalgia often suffer sleep disturbance, fatigue, cognitive impairment, depression and anxiety [1–4, 30]. Fibromyalgia reduces functioning in physical, psychological, and social spheres, and also has a negative impact on personal relationships, work, daily activities, and mental health [30]. Cold season seems to be associated with increased depression, [31, 32] although sometimes limited [33]. The scores of Hospital Anxiety and Depression Scale-Depression subscale were significantly higher in patients with weather sensitivity than those without weather sensitivity (Supplement Fig. 1.). Meanwhile, the associations among weather sensitivity, seasonality and other symptoms have not been shown in fibromyalgia.

Many patients report that weather sensitivity aggravates pain, [14–19] consistent with the present study. Although the pathologic mechanism behind the association is largely unknown, lowering barometric pressure induces neuronal activation in the superior vestibular nucleus in animals [34]. In a human study, air pressure is suggested to be associated with rheumatoid arthritis synovitis [35]. Meanwhile, the severity of symptom is associated with a belief about weather than in actual local weather situations [19]. Negative self-assessment has an important role in psychological disturbances, which are known to be associated with pain perception [36–38]. Psychological status may also partially mediate the association between weather sensitivity and pain; however, evidence on this is scarce [23, 24].

Interventions in combination and organization would benefit fibromyalgia [2–4]. Treatment plans should incorporate self-management techniques, goal-setting, and healthy lifestyle habits, acknowledging psychological distress when present. The effects of their treatment approaches on aggravating factors have not been shown.

There are several limitations to the present study. First, weather sensitivity was measured using only self-perceived symptoms using a binary scale, not a rating scale or actual local weather situations. Although the weather sensitivity using a binary scale is shown in one of the major aggravating factor in patients with fibromyalgia, [14, 16] there is a possibility of misclassification error. Second, the present study included only a small number of patients from a single medical center; our observations must therefore be interpreted with caution.

## Conclusions

Weather sensitivity, followed by pain intensity, was significantly associated with quality of life. There was an association with weather sensitivity and the minimal clinically important difference values of quality of life in patients with fibromyalgia. This suggests that weather sensitivity could play a key role in the quality of life in patients with fibromyalgia. Ascertaining the presence of weather sensitivity has the potential to be useful in estimating the level of suffering experienced by patients with fibromyalgia.

## Supplementary Information

**Additional file 1: Supplement Fig. 1.** Difference of HADS-Depression subscale between those with and without weather sensitivity. Values are means of the HADS-Depression subscale score, and the error bar shows standard deviations (*n* = 51). The HADS-Depression subscale scores in those with weather sensitivity were significantly worse than those without weather sensitivity. * Significance level was set at < 5%. HADS, Hospital Anxiety and Depression Scale.

## Data Availability

The datasets used and/or analysed during the current study are available from the corresponding author on reasonable request.

## Abbreviations

NRS: Numerical Rating Scale
EQ-5D-3L: Euro Quality of life-5 Dimensions-3 level
