# Supplementary material for: Weather sensitivity associated with quality of life in patients with fibromyalgia
Source: BMC Rheumatol. 2021 May 10;5:14. doi: 10.1186/s41927-021-00185-4 (PMC8108353; doi:10.1186/s41927-021-00185-4)
Supplement: Supplementary file 1 — Additional file 1: Supplement Fig. 1. Difference of HADS-Depression subscale between those with and without weather sensitivity. Values are means of the HADS-Depression subscale score, and the error bar shows standard deviations (n = 51). The HADS-Depression subscale scores in those with weather sensitivity were significantly worse than those without weather sensitivity. * Significance level was set at < 5%. HADS, Hospital Anxiety and Depression Scale. [file 41927_2021_185_MOESM1_ESM.pptx]

## Slide 1
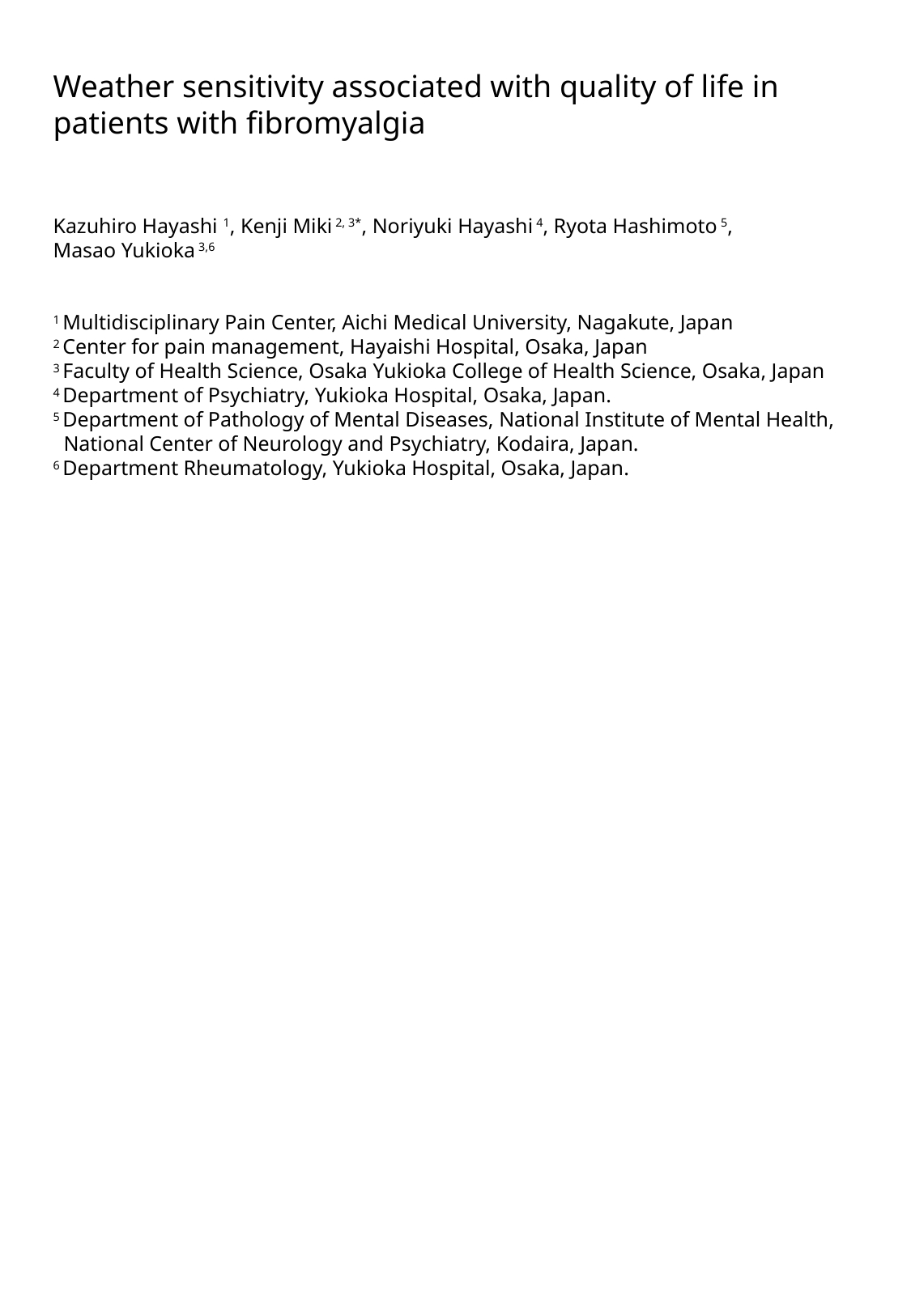

Weather sensitivity associated with quality of life in patients with fibromyalgia
Kazuhiro Hayashi 1, Kenji Miki 2, 3*, Noriyuki Hayashi 4, Ryota Hashimoto 5,
Masao Yukioka 3,6
1 Multidisciplinary Pain Center, Aichi Medical University, Nagakute, Japan
2 Center for pain management, Hayaishi Hospital, Osaka, Japan
3 Faculty of Health Science, Osaka Yukioka College of Health Science, Osaka, Japan
4 Department of Psychiatry, Yukioka Hospital, Osaka, Japan.
5 Department of Pathology of Mental Diseases, National Institute of Mental Health,
 National Center of Neurology and Psychiatry, Kodaira, Japan.
6 Department Rheumatology, Yukioka Hospital, Osaka, Japan.

## Slide 2
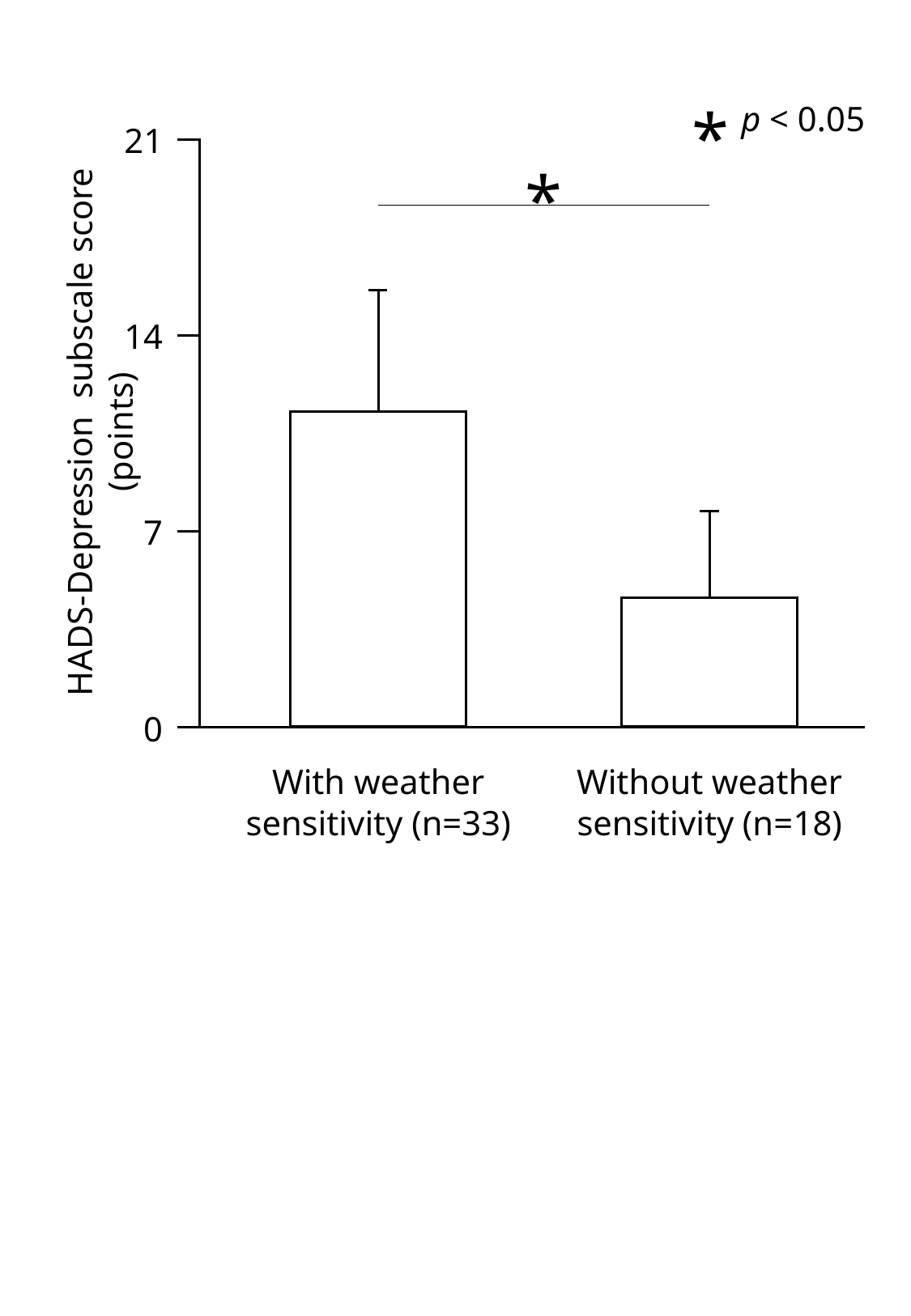

*
p < 0.05
21
*
14
HADS-Depression subscale score (points)
7
0
With weather
sensitivity (n=33)
Without weather
sensitivity (n=18)
